# The Effects of Proprioceptive Exercises on Postural Control in Handball Players with Chronic Ankle Instability—A Non-Randomized Control Trial

**DOI:** 10.3390/sports12110304

**Published:** 2024-11-11

**Authors:** Bogdan-Alexandru Antohe, Elena-Adelina Panaet

**Affiliations:** Faculty of Movement, Sports and Health Sciences, “Vasile Alecsandri” University of Bacau, Bacau 600115, Romania; antohe.bogdan@ub.ro

**Keywords:** ankle, sprain, center of pressure, instability, proprioception, handball

## Abstract

Background: This paper aims to investigate the impact of proprioceptive exercises on postural control in handball players with chronic ankle instability. Methods: The research participants (*n* = 22) were divided into two groups: the experimental group (*n* = 11) and the control group (*n* = 11). Chronic ankle instability was diagnosed using the Identification of Functional Ankle Instability (IdFAI) questionnaire, while postural control was evaluated with the Iso-Shift stabilometric platform. The intervention consisted of a 15-week proprioceptive exercise program, with sessions performed three times a week. The rehabilitation protocol was conducted at the start of each training session, immediately following the warm-up. Results: The data were analyzed using the Wilcoxon and Mann–Whitney U tests. Both groups improved their score on the Identification of Functional Ankle Instability (IdFAI) questionnaire (IdFAI_CG, *p* < 0.011; IdFAI_EG, *p* < 0.003) and reduced the number of ankle sprains (NS_EG, *p* < 0.008). Also, the experimental group had better results for the following tests: ellipse area with open eyes on the left leg (EA_I–OE_L, *p* < 0.009), ellipse area with closed eyes on the left leg (EA_I–CE_L, *p* < 0.033), anteroposterior deviation with open eyes on the left leg (APD_I–OE_L, *p* < 0.023), and the initial and final number of ankle sprains (NS_I, *p* < 0.01; NS_F, *p* < 0.024). Conclusions: Athletes who suffer from chronic joint instability are more likely to experience severe postural deviations than those who do not have this condition. Proprioceptive exercises had a positive impact on postural control in both groups, but the experimental group showed a greater improvement.

## 1. Introduction

Ankle sprains are one of the most common injuries among handball players [1]. About 70% of athletes who suffered an ankle sprain report chronic ankle instability (CAI) [2]. This condition is often characterized by pain during physical activity, sensations of “giving away”, and joint laxity [3,4,5].

Injuries occur due to quick changes in direction, cutting movements, or landings that force the foot into plantarflexion and inversion [6]. Other contributing factors include player contact during defense [7], along with fatigue, improper footwear, and unsuitable playing surfaces [8].

To maintain balance, the postural control system must work effectively in both unipedal and bipedal support, regardless of whether any factor could disrupt balance. The postural control system analyzes information received from various receptors, such as the vestibular, visual, and proprioceptive receptors, to produce an appropriate motor response that helps to control the body’s posture in different conditions, whether static or dynamic [9,10]. This feedback-based communication between the central nervous system and peripheral receptors ensures that the body can always adopt optimal postures. The literature reports that subjects with CAI have postural deficits [11,12,13,14].

There are various methods to evaluate postural control, but most studies focus on analyzing static and dynamic balance. Optimal communication between the central nervous system and peripheral sensory systems, as well as the anatomical integrity of the proprioceptive system (which includes ligamentous, capsular, and muscular receptors), is necessary for achieving optimal functioning of both the dynamic and static postural control system [15].

Athletes with a history of one or more ankle sprains and a diagnosis of chronic ankle instability (CAI) often experience changes in postural control due to altered sensory input from the proprioceptive receptors in their ankle joint to the central nervous system. Numerous research studies have confirmed these findings, highlighting various issues such as impaired neuromuscular coordination in the ankle, knee, and hip joints, stability deficits in the lumbopelvic and trunk regions, lumbar pain, and delayed activation of trunk muscles [16,17,18].

If athletes cannot maintain their center of gravity within the base of support, the literature recommends rehabilitation protocols based on proprioceptive and balance exercises [19]. Previous research demonstrates that this type of intervention is efficient in subjects with CAI, since it improves muscle spindle activity [20].

To our knowledge, no study has examined the effects of proprioceptive exercises on center-of-pressure distribution in handball players. Our research aims to highlight the differences in postural deviations between subjects with and without CAI and to demonstrate the efficiency of proprioceptive exercises in reducing these deviations.

## 2. Materials and Methods

### 2.1. Study Design

This study was carried out with a non-randomized control trial study design. According to this type of study, the investigator allocates participants into treatment and control groups [21]. We also followed the SPIRIT 2013 Checklist [22,23] and uploaded the database of the study on the Open Science Framework (OSF) platform (https://osf.io/v562k/ accesed on 4 November 2024).

### 2.2. Participants

The subjects were junior handball players (16.4 ± 0.52 years age) with a history of one or more ankle sprains. We used the following inclusion criteria: (1) age between 16 and 18 years, (2) at least three years of sports experience, and (3) the existence of one or more ankle sprains in the last year. The exclusion criteria were as follows: (1) 18+ years age, and (2) previous lower limb injury or surgery that could modify the results of balance tests.

**Figure 1:**
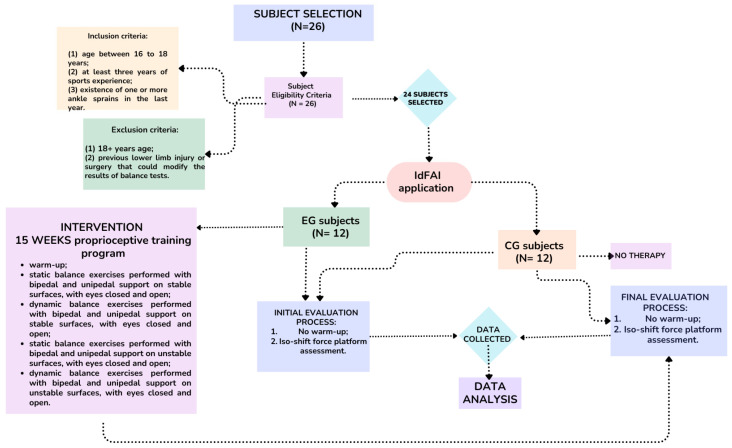
CONSORT flow diagram.

A total of 26 players were evaluated for eligibility, and 4 of them did not meet the inclusion/exclusion criteria. To classify the participants into control (GC) and experimental groups (GE), we used the Identification of Functional Ankle Instability (IdFAI) questionnaire, which is described in the assessment section. CG subjects (184.2 ± 9.5 cm height, 75.8 ± 9.4 kg weight) scored lower than 10 on the IdFAI questionnaire, while EG subjects (185.2 ± 4.85 cm height, 74.3 ± 9.8 cm) had a score higher than 10 (Figure 1).

### 2.3. Subject Assessment

This study employed a pre–post-trial design to evaluate the effectiveness of a proprioceptive training program over 15 weeks. The first assessment was conducted before the start of the proprioceptive training program, serving as a baseline measurement. The second assessment took place after 15 weeks of training, allowing for comparison with the baseline data (post-intervention). The participants were instructed to refrain from eating for at least two hours prior to the evaluation, and since they involved static activities, no warm-up was performed. Also, they were asked to stay barefoot and relaxed. The same evaluation protocol was used for both the baseline and post-intervention assessments to maintain consistency. The rehabilitation protocol was then carried out at the start of the training session, immediately following the warm-up.

#### 2.3.1. Iso-Shift Force Platform Assessment

Participants were assessed using the Iso-Shift force platform (TecnoBody, Bergamo, Italy). The Iso-Shift platform has previously been used to assess human posture in various occupational categories [24]. The platform is equipped with four pressure sensors that allow for real-time analysis of the center of gravity.

Before performing the tests, the participants were instructed by A.B.-A., a physiotherapist with experience of seven years. For each player, we tested the ankle-sprained leg. Usually, in handball players, the sprained ankle is opposite to the throwing arm. The participants performed three tests, with the first one being for familiarization with the platform, and the last two being for data collection. The best results achieved by the subjects were used for data analysis.

To assess the center of pressure (CoP) and trunk deviations, we chose the following tests:

The stability test consists of two subtests: the first one measures bipedal balance with eyes open, while the second one measures bipedal balance with eyes closed. To perform the test, the athlete stands on the platform with his arms crossed at chest level and feet together, respecting the landmarks indicated by the platform. To perform the test, the subject has to maintain this position for 30 s. When the participant is ready, the operator presses the “start” button, and the test begins. The subjects did not receive any verbal or auditory feedback during testing.

The stability test on the left leg follows the stability test protocol, with the difference being that the assessment is carried out with unipedal left leg support.

The stability test on the right leg follows the stability test protocol, with the difference being that the assessment is carried out with unipedal right leg support.

#### 2.3.2. Identification of Functional Ankle Instability (IdFAI) Questionnaire

The IdFAI questionnaire is a reliable tool to measure ankle instability in athletes. It consists of ten questions, each with a scale from 0 to 5, that indicates the severity of the athlete’s symptoms. The IdFAI questionnaire can be seen in the study of Simon et al., 2013 [25], where a higher score on the questionnaire indicates a greater degree of joint instability. The questionnaire has a sensitivity of 89.6% [25] and a reliability between 0.92 and 0.97 [26]. We used the IdFAI questionnaire to create both experimental and control groups. The experimental group had higher scores on the questionnaire, indicating higher levels of ankle instability.

### 2.4. Intervention

We conducted a 15-week proprioceptive training program focused on the non-dominant leg (the leg opposite the throwing arm) at the beginning of each training session. Each session lasted approximately 15 min and was held three times per week. The 15-week duration was selected based on research indicating that ankle instability and postural control improvements typically emerge after 12 weeks of training [27,28,29]. For exercise progression, we followed the recommendations of Borreani [30]. The exercises we applied were as follows: static balance exercises performed with bipedal and unipedal support on stable surfaces, with eyes closed and open; dynamic balance exercises, like the Y balance and Star Excursion Exercise, performed with bipedal and unipedal support on stable surfaces, with eyes closed and open; static balance exercises performed with bipedal and unipedal support on unstable surfaces, with eyes closed and open; dynamic balance exercises performed with bipedal and unipedal support on unstable surfaces, with eyes closed and open.

## 3. Results

### 3.1. Statistical Analysis

Prior to data analysis, we conducted an Independent T-Test to compare the demo-graphics of participants (height, weight, and age). The results showed no significant differences (Table 1). For further analysis, we used two non-parametric statistical tests: the Wilcoxon and Mann–Whitney U tests. The Wilcoxon test was applied to emphasize the differences between the initial and final scores of the same group (control or experimental), while the Mann–Whitney U test was used to reveal the differences between the two groups [31]. We calculated the effect size according to Cohen’s instructions, where r = 0.20 indicates a small effect size, r = 0.50 indicates a medium effect size, and r = 0.80 indicates a large effect size [32].

### 3.2. The Wilcoxon Test Results

Table 2 shows the results obtained by the two samples (control and experimental groups) after applying the non-parametric Wilcoxon test.

Table 2 indicates a statistically significant difference between the initial and final assessments (in both groups) for a significant number of parameters: IdFAI_CG, *p* < 0.011; IdFAI_EG, *p* < 0.003; NS_EG, *p* < 0.008; EA, P, APD, MLD, and SDT. The obtained results highlight the major impact of the proprioceptive exercise program on the qualitative movement parameters (IdFAI values, number of ankle sprains, trunk and CoP deviations in unipedal and bipedal support, with eyes closed and open).

Table 3 shows the comparative values between the two samples (experimental and control groups) before and after applying the proprioceptive exercise program.

Based on the Mann–Whitney U test results, there are significant differences between the initial assessment of the experimental and control groups. This is seen in the following parameters: IdFAI_I with *p* < 0.001, IdFAI_F with *p* < 0.001, EA_I–OE_L with *p* < 0.009, EA_I–CE_L with *p* < 0.033, APD_I–OE_L with *p* < 0.023, NS_I with *p* < 0.01, NS_F with *p* < 0.024, and MLD_I–CE_L with *p* < 0.023. The final assessment showed no discernible statistical differences between the two groups, suggesting normalization of the results. Notably, the experimental group demonstrated improved outcomes, which were confirmed by the results of the Wilcoxon test. After comparing the functional parameter values between the initial and final assessments of both groups, it is evident that there was an improvement in all measured parameters, especially for the experimental group. The effect size for the parameters that recorded statistically significant values was as follows: IdFAI_I, r = 0.73; IdFAI_F, r = 0.66; EA_I–OE_L, r = 0.32; EA_I–CE_L, r = 0.21; APD_I–CE_L, r = 0.24; NS_I, r = 0.65; NS_F, r = 0.24; MLD_I–CE_L, r = 0.24.

## 4. Discussion

CAI is one of the most common consequences of a sprained ankle. This condition modifies the lower limb muscle contraction pattern [33] and ankle stability [34]. Over time, muscle imbalances can reach the trunk and gluteal muscles [35]. Based on our research analysis, it can be confirmed that individuals with CAI demonstrated greater trunk deviations compared to those without CAI. The most significant difference was observed on the leg with the ankle sprain (the left leg), with eyes closed (P_CE–EG, Z = −2.312, *p* < 0.021).

Our CAI group exhibited higher levels of trunk deviation during unipedal balance tests, especially in the anteroposterior (APD_I–OE_L, *p* < 0.023) and mediolateral (MLD_I–CE_L, *p* < 0.023) directions, regardless of whether the eyes were open or closed. Many studies reported changes in the CoP direction, whether it be anterior [36], posterior, or mediolateral [37,38], in subjects with chronic ankle instability (CAI) during unipedal stance tests. Based on our research and the literature, we can confirm that each participant has a unique alteration in their CoP. There are a few factors that may contribute to the direction of the compensatory mechanism: (i) a sprained ankle due to an inversion mechanism (mediolateral deviations), (ii) restricted dorsiflexion range of motion (anteroposterior deviations), and (iii) a supraspinal adaptation mechanism of the postural control system that aims to protect the lower extremity [13].

Individuals with chronic joint instability often experience unstable joints and tend to counterbalance this by “locking” their ankle joint to gain stability [39]. However, this lack of mobility in the ankle joint results in modifying the adaptation strategy of the postural system, from ankle to hip strategy, which can significantly modify CoP values. This compensatory mechanism provides a rationale for the increased CoP values in people with CAI.

One effective way to improve the postural control system is by performing proprioceptive exercises. During our research, we followed the progressive training protocol as described in the literature [40,41,42]. At the end of the experimental period, proprioceptive exercises proved their effectiveness by improving all tested functional parameters and ultimately by reducing the number of sprains.

In the control group, from an initial number of four sprains, at the end of the experiment, only one athlete experienced another ankle sprain, indicating that the number of ankle sprains decreased by 75%. In the experimental group, there were 39 ankle sprains at the beginning of the study. At the end of the study, only eight ankle sprains were reported by the athletes, indicating a decrease of 80.5% in the number of ankle sprains. The initial (*p* < 0.001) and final (*p* < 0.024) assessments indicate a significant difference in the number of ankle sprains between the two groups. The experimental group had a much smaller number of ankle sprains compared to the control group.

Other studies have shown a significant reduction in the incidence of ankle sprains. McGuine [43] reported a 47% reduction, Hupperets [44] reported a 35% reduction, and Bahr [45] reported a 50% reduction. However, in these studies, the follow-up period was longer than the one used in our research. We only counted ankle sprains during the experiment, which is a limitation of our study. If we had extended the follow-up period, we believe that the number of ankle sprains would have been higher.

The implementation of a proprioceptive exercise program for the participants included in the experiment produced an improvement in joint stability, which in turn improved ankle joint mobility and postural control system function. All of these positive changes can be attributed to an increase in neuromuscular reactivity through the recruitment of neuromuscular spindles [46,47], as well as an increase in the number of sensory inputs to the central nervous system [48]. Additional sensory inputs allow for better organization of the postural control system and therefore an improvement in CoP values for players with chronic joint instability. In our research, the experimental group achieved significantly better values in the final tests performed with their eyes closed. These results reveal the importance of sensory feedback for CoP normalization and confirm the idea that individuals with chronic joint instability show significant deficits when tested for unipedal balance with eyes closed [49].

After conducting our research, we found that all of the subjects demonstrated an improvement in their CoP values across all tests performed. These findings emphasize the effectiveness of the proprioceptive exercise program on trunk deviation in individuals with CAI. Our results are supported by research reports from [49,50,51].

The clinical relevance of this study results from the normalization of CoP values. The better the CoP values, the lower the risk of injury. This aspect is even more important in handball games, which is a contact sport and requires many jumps [52]. To minimize the risk of injury, the optimal distribution of the center of gravity during ground contact is particularly important [53].

The normalization of differences in balance values between the two groups (highlighted in Table 3) during the initial assessment indicates greater progress achieved by the experimental group. However, we emphasize that in the initial assessment, these statistically significant differences were obtained only for the left leg, namely the leg with chronic joint instability.

Comparing the results of the two groups, we can therefore note that more progress is made by the experimental group in the final assessment (with the statistical significance threshold being exceeded in all balance tests; see Table 2). This indicates that individuals with chronic joint instability make greater progress after proprioceptive exercises than those without chronic joint instability.

### Study Limitations, Strengths, and Weaknesses

Our study’s limitation stems from the use of purposive sampling. Although it enabled us to focus on specific characteristics, it led to a relatively small sample size, potentially reducing the generalizability of our results to a wider population of handball players, especially those with different age ranges or injury backgrounds. Another limitation of our study is the follow-up period, which could have been longer to better assess the long-term effects of proprioceptive exercises.

While purposive sampling may limit broader generalizability, it can be a strength when focusing on specific groups like junior handball players, as it allows for a deeper exploration of chronic ankle instability. Moreover, the combination of the IdFAI questionnaire with balance tests using advanced equipment offers a robust approach to evaluating ankle functional stability.

The weakness of our study is represented by the potential selection bias. The specific inclusion and exclusion criteria may lead to selection bias, as they limit the diversity of the participant pool.

In our future research plans, we propose to address these limitations and expand our understanding of chronic ankle instability in athletes. We want to establish a larger sample size, conducting studies with larger and more diverse populations to enhance the generalizability of findings across different age groups and sports disciplines. Also, we want to track the progression of balance and stability issues over time, among the control group, examining how chronic ankle instability impacts their long-term athletic performance.

## 5. Conclusions

Both the experimental and control group demonstrated improvements in center-of-pressure (COP) distribution as a result of proprioceptive exercises, with the experimental group showing superior outcomes. The normalization of COP distribution contributed to a reduced incidence of sprains among players during the intervention period. Therefore, proprioceptive exercises can be considered an effective preventive strategy against ankle sprains.

## Figures and Tables

**Table 1:** Participant demographics.

Measurement	CG		EG	
	I	F	I	F
H (m)	184.2 ± 9.5	186.2 ± 8.94	185.2 ± 4.85	187.5 ± 5.88
W (kg)	75.8 ± 9.4	79 ± 10.4	74.3 ± 9.8	77.4 ± 10.4
A (years)	16.4 ± 0.52		16.4 ± 0.52	

Note: H = height, W = weight, A = age, I = initial value, F = final value, CG = control group, and EG = experimental group.

**Table 2:** Wilcoxon test results. Comparative values within same group.

Item No.	Variable		Z (Wilcoxon)		*p*		Effect Size (r)	
			CG	EG	CG	EG	CG	EG
1	IdFAI		−2.536	−2.937	0.011	0.003	−0.54	−0.62
2	NS		−1.732	−2.673	0.083	0.008	-	−0.59
3	EA	OE	−2.312	−2.934	0.021	0.003	−0.51	0.65
		CE	−1.876	−1.600	0.062	0.110	-	-
		OE_L	−2.934	−2.756	0.003	0.006	−0.62	−0.58
		OE_R	−2.312	−2.756	0.021	0.006	−0.49	−0.58
		CE_L	−2.845	−2.934	0.004	0.003	−0.60	−0.62
		CE_R	−2.845	−2.934	0.004	0.003	−0.60	−0.62
4	P	OE	−0.533	−2.045	0.594	0.041	-	−0.45
		CE	−1.067	−2.312	0.286	0.021	-	−0.49
		OE_L	−0.445	−1.867	0.657	0.062	-	-
		OE_R	−0.445	−2.490	0.657	0.013	-	0.61
		CE_L	−2.934	−2.934	0.003	0.003	−0.60	−0.60
		CE_R	−2.756	−2.756	0.006	0.006	−0.62	−0.58
5	APD	OE	−2.756	−2.934	0.006	0.003	−0.58	0.60
		CE	−2.045	−0.356	0.041	0.722	−0.43	-
		OE_L	−1.956	−2.934	0.050	0.003	−0.41	−0.60
		OE_R	−0.899	−1.778	0.374	0.075	-	-
		CE_L	−2.934	−2.934	0.003	0.003	−0.62	−0.62
		CE_R	−2.845	−2.587	0.004	0.010	−0.60	−0.55
6	MLD	OE	1.689	−2.934	0.091	0.003	-	−0.62
		CE	−1.156	−1.956	0.248	0.05	-	−0.41
		OE_L	−2.667	−2.312	0.008	0.021	−0.56	−0.49
		OE_R	−2.401	−2.312	0.016	0.021	−0.51	−0.49
		CE_L	−2.845	−2.934	0.004	0.003	−0.60	−0.62
		CE_R	−2.934	−2.934	0.003	0.003	−0.62	−0.62
7	SDT	OE	1.334	−2.601	0.182	0.009	-	−0.58
		CE	−0.89	−0.624	0.929	0.533	-	-
		OE_L	−0.622	−1.112	0.534	0.266	-	-
		OE_R	−1.824	−0.978	0.068	0.328	-	-
		CE_L	−0.267	−2.312	0.790	0.021	-	−0.49
		CE_R	−1.778	−1.778	0.075	0.075	-	-

Note: IdFAI = Identification of Functional Ankle Instability questionnaire, NS = number of sprains, EA = ellipse area, P = perimeter, APD = anteroposterior deviation of the trunk, MLD = mediolateral deviation of the trunk, SDT = standard deviation of the trunk, OE = open eyes, and CE = closed eyes.

**Table 3:** The Mann–Whitney U test values. Comparing the values between the control and experimental groups.

Item No.	Variable		Z (Mann–Whitney U)	*p*	Effect Size (r)	Item No.	Variable		Z (Mann–Whitney U)	*p*	Effect Size (r)
1	IdFAI_I		−3.920	0.001	0.73	8	NS_I		−3.701	0.001	0.65
2	IdFAI_F		−3.736	0.001	0.66	9	NS_F		−2.256	0.024	0.24
3	EA_I	OE	−0.230	0.818	-	10	APD_F	OE	−1.149	0.250	-
		CE	−0.624	0.533	-			OC	−0.066	0.948	-
		OE_L	−2.594	0.009	0.32			OE_L	−0.098	0.922	-
		OE_R	−0.361	0.718	-			OE_R	−0.472	0.670	-
		CE_L	−2.134	0.033	0.21			OC_L	−0.624	0.533	-
		CE_R	−0.558	0.577	-			OC_R	−1.740	0.082	-
4	EA_F	OE	−0.624	0.533	-	11	MLD_I	OE	−0.558	0.577	-
		CE	−0.723	0.470	-			OC	−0.361	0.718	-
		OE_L	−0.098	0.922	-			OE_L	−1.740	0.082	-
		OE_R	−0.624	0.533	-			OE_R	−0.427	0.670	-
		CE_L	−1.149	0.250	-			OC_L	−2.264	0.023	0.24
		CE_R	−1.937	0.053	-			OC_R	−0.624	0.533	-
5	P_I	OE	−0.492	0.622	-	12	MLD_F	OE	−0.624	0.533	-
		CE	−0.98	0.922	-			OC	−0.887	0.375	-
		OE_L	−1.871	0.061	-			OE_L	−1.215	0.224	-
		OE_R	−0.886	0.375	-			OE_R	−1.543	0.123	-
		CE_L	−0.886	0.375	-			OC_L	−1.117	0.264	-
		CE_R	−0.295	0.768	-			OC_R	−1.248	0.212	-
6	P_F	OE	−1.148	0.250	-	13	SDT_I	OE	−0.788	0.430	-
		CE	−1.477	0.140	-			OC	−0.526	0.599	-
		OE_L	−0.098	0.922	-			OE_L	−1.544	0.123	-
		OE_R	−1.083	0.279	-			OE_R	−1.413	0.158	-
		CE_L	−0.230	0.818	-			OC_L	−0.821	0.412	-
		CE_R	−1.871	0.061	-			OC_R	−0.427	0.670	-
7	APD_I	OE	−0.164	0.870	-	14	SDT_F	OE	0.493	0.622	-
		CE	−1.609	0.108	-			OC	−0.460	0.645	-
		OE_L	−2.265	0.023	0.24			OE_L	−0.788	0.431	-
		OE_R	−0.558	0.577	-			OE_R	−0.493	0.622	-
		CE_L	−1.415	0.158	-			OC_L	−0.361	0.718	-
		CE_R	−0.886	0.375	-			OC_R	−0.624	0.533	-

Note: IdFAI = Identification of Functional Ankle Instability questionnaire, NS = number of sprains, EA = ellipse area, P = perimeter, APD = anteroposterior deviation of the trunk, MLD = mediolateral deviation of the trunk, SDT = standard deviation of the trunk, OE = open eyes, and CE = closed eyes.

## Data Availability

The original data presented in the study are openly available in Open Science Framework at https://osf.io/v562k/ (accessed on 4 November 2024).
